# Rheological and Microstructural Characterization of Novel High-Elasticity Polymer Modifiers in Asphalt Binders

**DOI:** 10.3390/polym17192704

**Published:** 2025-10-08

**Authors:** Syed Khaliq Shah, Ying Gao, Abdullah I. Almansour

**Affiliations:** 1 School of Transportation, Southeast University Road, Jiangning District, Nanjing 211189, China; 233209946@seu.edu.cn; 2 Civil Engineering Department, King Saud University, Riyadh 11451, Saudi Arabia

**Keywords:** high-elasticity polymer modifiers, viscoelastic behavior, complex modulus, rheological behavior, master curve analysis

## Abstract

This study investigates the rheological, thermal, and microstructural performance of three novel high-elasticity polymer modifiers (HEMs) incorporated into asphalt binders. The modifiers were evaluated at their recommended dosages using a multi-scale framework combining rotational viscosity, dynamic shear rheometry (frequency sweeps, Cole-Cole plots, Black diagrams, and master curves), bending beam rheometry, differential scanning calorimetry (DSC), fluorescence microscopy (FM), atomic force microscopy (AFM), and Fourier transform infrared spectroscopy (FTIR). Results show that HEM-B achieved the highest values of the superpave rutting parameter (G*/sinδ = 5.07 kPa unaged, 6.73 kPa aged), reflecting increased high-temperature stiffness but also higher viscosity, which may affect workability. HEM-C exhibited the lowest total enthalpy (1.18 W·g^−1^) and a glass transition temperature of −7.7 °C, indicating improved thermal stability relative to other binders. HEM-A showed the greatest increase in fluorescent area (+85%) and the largest reduction in fluorescent number (−60%) compared with base asphalt, demonstrating more homogeneous phase dispersion despite higher enthalpy. Comparison with SBS confirmed that the novel HEMs not only meet but exceed conventional performance thresholds while revealing distinct modification mechanisms, dense cross-linking (HEM-B), functionalized thermoplastic compatibility (HEM-C), and epoxy-tackified network formation (HEM-A). These findings establish quantitative correlations between rheology, thermal stability, and microstructure, underscoring the importance of dosage, compatibility, and polymer network architecture. The study provides a mechanistic foundation for optimizing high-elasticity modifiers in asphalt binders and highlights future needs for dosage normalization and long-term aging evaluation.

## 1. Introduction

Asphalt pavements form the backbone of modern highway infrastructure, yet their mechanical and thermal performance remains highly vulnerable to service conditions. At elevated temperatures, conventional asphalt softens, leading to rutting and permanent deformation, while at low temperatures it hardens and becomes brittle, causing thermal cracking. The combined effects of traffic loading, multi-axle vehicles, and harsh climatic conditions accelerate binder aging and shorten pavement service life. To overcome these shortcomings, polymer modification has been widely applied as one of the most effective approaches to improve elasticity, durability, and overall binder stability.

Among the various modifiers, styrene butadiene styrene (SBS) has been most extensively used due to its excellent elasticity and rutting resistance [1]. However, SBS suffers from phase separation and poor storage stability, particularly under high-temperature conditions [2]. Other polymers such as ethylene–vinyl acetate (EVA) enhance stiffness but demonstrate poor compatibility with asphalt matrices, while crumb rubber improves elasticity and sustainability but requires high processing temperatures and presents dispersion challenges. Epoxy resins offer significant rutting resistance but often lead to brittleness at low temperatures. These limitations highlight that although polymer modification provides performance benefits, no single modifier offers a universal solution [3,4].

To address these shortcomings, high-elasticity polymer modifiers (HEMs) have been developed as advanced alternatives. These modifiers are designed to improve elastic recovery, rutting resistance, and binder durability across a broad temperature range [5,6]. Previous studies report that HEMs can enhance cohesion, structural stability, and moisture resistance in dense-graded asphalt concrete (AC-16) and stone mastic asphalt (SMA-16) mixtures [7]. However, when applied to SBS-modified binders, their incremental benefits appear limited because the existing polymer network already provides substantial elasticity and deformation resistance [8]. This binder dependency raises critical questions regarding the compatibility of HEMs with conventional polymer-modified asphalts, which remain largely unanswered in the current literature.

Mechanistic investigations have demonstrated that elastomeric polymers improve elastic recovery, typically confirmed by conventional penetration, softening point, and elastic recovery tests [9]. More advanced characterization using dynamic shear rheometer (DSR) testing has enabled a deeper understanding of viscoelastic behavior under varying conditions, with instantaneous and delayed moduli identified as reliable indicators of elastic capacity. The rheological properties of asphalt binders are closely linked to their microstructure and the dispersion of their constituents. The rheological properties of asphalt binders are closely linked to their microstructure and the dispersion of their constituents [10,11,12]. Physically co-blended modified binders typically exhibit two distinct domains: a swollen polymer-rich phase and the surrounding asphalt binder matrix, each contributing uniquely to overall performance [13].

Polyurethane prepolymers have also been shown to enhance high-temperature stability and compatibility through micro-network formation and secondary bonding interactions that reduce phase separation. However, many of these findings remain descriptive, reporting performance improvements without adequately explaining the chemical or microstructural mechanisms responsible [3,14]. Recent studies have explored polymer modifiers with chemical features analogous to those being investigated in this study. For instance, Ref. [15] developed SBS/epoxy hybrid binders, where a mono-component epoxy was grafted onto SBS to enhance high-temperature stability. Their results showed improved elastic recovery, but also highlighted challenges in storage stability due to premature crosslinking limitations commonly observed with polymer blends designed to improve asphalt performance [16,17]. Thermoset-resin-based anti-rutting additives, such as polyurethane prepolymers and high-modulus agents, which form dense three-dimensional (3D) networks, have also been evaluated. These systems demonstrated superior rutting resistance, but at the cost of compromised low-temperature flexibility, a trade-off often encountered with polymer-modified asphalts aimed at addressing high-temperature deformation. Furthermore, CO_2_ based polyurethane-modified asphalts, which rely on polar interactions and physical entanglement for compatibility within the asphalt matrix, have been investigated. This mechanism is like the functionalized thermoplastic design used in modern polymer modifiers. While these studies confirm the performance potential of chemically tailored modifiers, none provide a multi-scale correlation linking rheology, thermal behavior, and microstructure, as performed here [18].

Previous research has consistently demonstrated that elastomeric polymers enhance the elastic response and recovery capacity of asphalt binders [19]. This improvement is typically verified through conventional tests and elastic recovery, all of which indicate that the addition of elastomeric materials leads to a stiffer and more resilient binder structure [20]. Despite these advances, asphalt binders remain susceptible to both physical and chemical degradation during their service life. Oxidative aging leads to the volatilization of light fractions, an increase in carbonyl/sulfoxide content [21], and progressive hardening, which ultimately results in embrittlement and thermal cracking. To mitigate these effects, various strategies such as antioxidant additives, anti-stripping agents, and rejuvenators including bio-oils and waste plastics have been explored to restore flexibility and extend the lifespan of pavements. However, the compatibility of these strategies with advanced polymer systems, particularly high-elasticity modifiers (HEMs), remains poorly understood. While HEMs enhance the initial performance of asphalt binders, their long-term aging resistance and potential for rejuvenation in recycled asphalt pavements (RAP) have not been systematically evaluated. This knowledge gap limits the ability to deploy HEMs sustainably within circular economy frameworks [22].

In China, several innovative high-elasticity modifiers have already been applied in practice, developed by leading polymer research institutes and transportation technology centers. Their successful use on high-grade highways such as G235, S341, and S356 demonstrates the feasibility of incorporating HEMs into asphalt mixtures, even though detailed academic studies on their mechanisms remain limited.

The present study addresses these gaps by investigating three novel HEMs with distinct chemical strategies: HEM-A, HEM-B and HEM-C. A comprehensive framework is applied that integrates rheological characterization, thermal stability analysis (differential scanning calorimetry, DSC), and microstructural evaluation (FM, AFM, and FTIR). This approach seeks to clarify the mechanisms of modification, establish correlations across multiple scales, and provide practical insights for the design of durable, high-performance asphalt binders.

## 2. Materials and Methods

### 2.1. Raw Materials

#### 2.1.1. Asphalt

The physical properties of the base asphalt (BA), including penetration, softening point, and viscosity, were determined by standard test methods ASTM D3381-21 [23], as summarized in Table 1.

#### 2.1.2. Modifier

**Figure 1:**
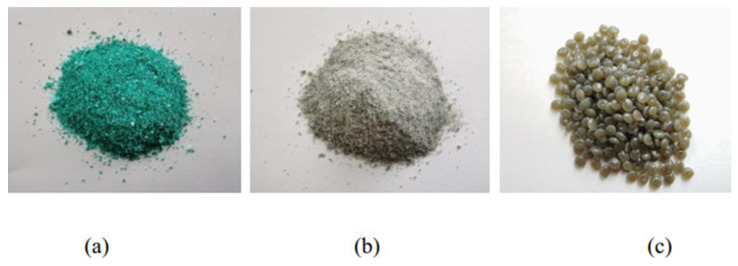
Appearance of high-elasticity polymer modifiers used in the study: (**a**) HEM-A, (**b**) HEM-B, (**c**) HEM-C.

Three types of high-elasticity polymer modifiers (HEMs), namely HEM-A, HEM-B, and HEM-C, were utilized in this study, as shown in Figure 1, to investigate their distinct influences on asphalt binders. These modifiers were selected based on their advanced formulation chemistry and potential to enhance the high-temperature performance and elasticity. HEM-A is an SBS based matrix grafted with a mono component epoxy and ractive tackifier. The epxoy group introduce additional cross-linking sites, while the tackifier improves interfacial bonding between the polymer and asphalt, enhancing elasticity and dispersion.

HEM-B is a thermosetting polymer blended with a synthetic resin that yields a dense cross-linked network. The high crosslink density restricts molecular chain mobility, resulting in greater stiffness and viscosity but reduced low-temperature flexibility.

HEM-C is a thermoplastic polymer functionalized with linear graft groups and compounded with compatibilizing additives. This structure promotes entanglement and the formation of a spatial polymer network, providing superior compatibility and thermal stability. The differing chemical compositions of HEM-A (epoxy-tackified SBS), HEM-B (thermoset–resin blend), and HEM-C (functionalized thermoplastic) are critical in explaining their rheological and microstructural performance differences. Table 2 summarizes their fundamental physical properties and chemical composition. The HEMs were sourced from Jiangsu Jicui Advanced Polymer Materials Research Institute, Guolu Gaoke Engineering Technology Institute Co., Ltd., and Jiangsu Transportation Technology Institute Co., Ltd.

### 2.2. Preparation of Specimens

High-elasticity polymer modifiers (HEM-A, HEM-B, and HEM-C) were incorporated into the asphalt binder at a dosage of 10 wt% in accordance with manufacturer recommendations, based on the total mass of the binder. For comparison, SBS was incorporated at 4.5 wt%, following the conventional dosage specified in ASTM D6114-97 [24] for SBS-modified asphalt. These dosage levels reflect standard practice and supplier guidance for each modifier system.

**Figure 2:**
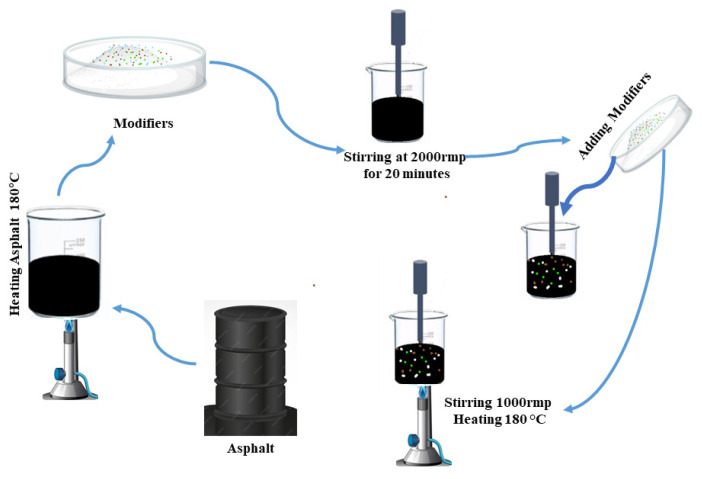
Preparation process for asphalt binders modified with high-elasticity polymer modifiers (HEMs).

For comparative purposes, an SBS-modified asphalt binder (pure bitumen) was prepared using a conventional dosage of 4.5 wt%, by standard polymer modification practices outlined in ASTM D6114-97 [24]. Approximately 400 (g) of base asphalt was placed in a jacketed vessel and heated to a constant temperature of 180 (°C). It was weighed according to the required mass and then gradually added to the molten asphalt in 5–8 separate portions to ensure uniform blending. The blending process was carried out using a high-shear mixer (FLUKO FM30D, Germany) operating at 2000 rpm for 20 min with a rotor stator head. To ensure mixture homogeneity, dispersion was monitored by observing torque stability during mixing and visually checking the absence of unblended particles. Following this initial high-shear stage, the temperature was maintained at 180 °C, and the blend was further mixed with a mechanical stirrer at 1000 rpm for an additional 20 min. The ratio of mixer radius to vessel radius was approximately 0.45, which ensured sufficient shear coverage inside the vessel. This two-stage mixing process was designed to achieve uniform integration of the modifiers and improve the overall homogeneity of the asphalt binder. The preparation process is illustrated in Figure 2 [25].

### 2.3. Experimental Methods

#### 2.3.1. Rotational Viscosity (RV) Test

The workability and flow behavior of the asphalt binders at high temperatures were assessed using a rotational viscosity test, performed by the AASHTO T 316-21 standard [26]. A Brookfield rotational viscometer fitted with spindle No. 27 was used for this test. The binder samples were tested at three temperatures: 130 (°C), 150 (°C), 165 (°C), and 180 (°C). For each temperature, viscosity measurements were recorded at rotational speeds of 20 rpm and 50 rpm. The binder samples were preheated to ensure homogeneity and free flow. Each sample was then transferred into the viscometer container and conditioned at the test temperature for 10 min to achieve thermal equilibrium. Viscosity values were recorded once stable readings were observed. Each test was repeated three times at each temperature to ensure repeatability, and the average viscosity values, along with their standard deviations, were calculated.

#### 2.3.2. Bending Beam Rheometer (BBR)

The low-temperature rheological behavior of asphalt binders modified with high-elasticity modifiers (HEMs) was characterized using the Bending Beam Rheometer (BBR) by AASHTO T 313 [27,28]. This procedure evaluates the binder’s resistance to thermal cracking by measuring creep stiffness (S) and creep rate (m) under constant load and low-temperature conditions.

Beam specimens (127 mm × 12.7 mm × 6.25 mm) were conditioned at target temperatures for 60 min before testing. Creep tests were conducted at temperatures ranging from 24 °C to 12 °C in 3 °C increments under a constant load of 980 ± 50 mN for 240 s, with continuous mid-span deflection monitoring. Each test was performed in triplicate, and results are reported as mean ± standard deviation. Creep stiffness (S) and m-value were calculated to evaluate low-temperature performance. Stress (σ) was defined as applied load per unit area, while strain (ε) represented deformation under load. The time-dependent stress–strain relationship facilitated the determination of S and m-value, indicating resistance to thermal cracking.

#### 2.3.3. Frequency Sweep Rheological Test

The rheological properties of modified asphalt binders were evaluated using a Dynamic Shear Rheometer (DSR) by AASHTO T 315-21. Frequency sweep tests were performed over a range of 0.1–10 Hz at temperatures from 40 °C to 75 °C, in 10 °C increments. An 8 mm parallel plate geometry with a 2 mm gap was used for testing at temperatures below 40 °C. The complex shear modulus and phase angle were recorded at each frequency to characterize the viscoelastic behavior of the binders. Master curves were developed using the time-temperature superposition principle (TTSP) with 20 °C as the reference temperature. The individual isothermal frequency sweep data at each test temperature were horizontally shifted along the frequency axis until continuity was achieved.

The horizontal shift factors (*_a_*T) were determined using the Williams–Landel–Ferry (WLF) Equation (1).

(1) $$logaT=\frac{c_{1}(T-T_{r})}{C_{2}+(T-T_{r})}$$

where *T* is the test temperature (°C), *T_r_* is the reference temperature (60 °C), and *C*1 and *C*2 are empirical constants obtained by fitting the experimental data. The reduced frequency (*f* × *aT*) was then used as the horizontal axis to construct the master curves for both |G*| and δ. The calculated shift factors for each test temperature are summarized in Table 3.

#### 2.3.4. Fourier Transform Infrared Spectroscopy Test (FTIR)

Fourier Transform Infrared (FTIR) spectroscopy was performed using a Bruker spectrometer to identify the functional groups present in the samples. Spectra were recorded in the range of 4000–400 cm^−1^ using attenuated total reflectance (ATR) mode. Each spectrum was obtained by averaging 32 scans per sample at a resolution of 4 cm^−1^. The resulting absorption peaks were analyzed to confirm the presence of specific chemical functionalities and to assess any structural changes occurring in the modified materials.

#### 2.3.5. Fluorescence Microscopy Test

Fluorescence microscopy (FM) was conducted to investigate the microstructural dispersion and compatibility of high-elasticity modifiers (HEMs) within the asphalt binder. Thin films of the modified binder were prepared by heating to 140–150 °C and then deposited on glass slides. After surface smoothing and cooling to room temperature, samples were examined under ultraviolet (UV) illumination. The swollen polymer phase fluoresced distinctly due to its absorption of light oil fractions in the asphalt matrix. Micrographs were captured at 100× magnification, where bright regions indicated domains rich in modifiers. Multiple fields of view were analyzed to ensure representative and consistent results.

#### 2.3.6. Atomic Force Microscope (AFM) Test

Atomic Force Microscopy (AFM) was employed to investigate the nanoscale surface morphology of modified asphalt binders. Samples were heated to a flowable state at 160 ± 5 °C and uniformly spread onto clean glass slides, followed by oven treatment for 5 min to achieve a smooth surface finish. After cooling to ambient temperature, AFM imaging was performed in tapping mode at 25 °C over a 20 μm × 20 μm scan area. Height images were obtained from at least three different regions of each specimen to ensure data consistency. The topographical analysis provided insights into surface roughness, microstructural uniformity, and phase distribution of the high-elasticity modifiers within the asphalt matrix.

## 3. Results

### 3.1. Brookfield Viscosity Analysis

**Figure 3:**
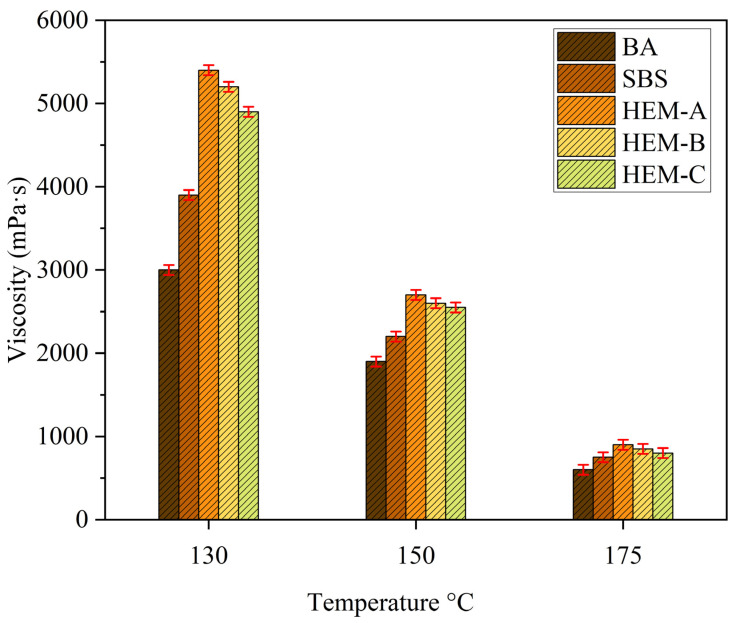
Results of rotational viscosity test.

Figure 3 presents the Brookfield viscosity profiles of the base asphalt (BA) and its modifications with HEM-A, HEM-B, and HEM-C. Viscosity is a critical rheological parameter for evaluating the high-temperature performance and workability of asphalt binders. As expected, all binders exhibited a consistent decrease in viscosity with increasing temperature, reflecting typical thermorheological behavior. The incorporation of HEM-A, HEM-B, and HEM-C into BA significantly increased viscosity across the entire temperature range tested, indicating improved resistance to flow and thermal deformation. The viscosities of the HEM-modified binders exceeded those of the SBS-modified binder by a factor of 1–2, highlighting the superior stiffening effect of the HEMs. Among the evaluated binders, HEM-B exhibited the highest viscosity at most temperatures, followed by HEM-A, HEM-C, SBS, and BA.

However, the elevated viscosity associated with HEM-B may adversely affect the workability of the binder, necessitating higher mixing and compaction temperatures. This could lead to increased energy consumption during construction and operational challenges. Additionally, at 130 °C, the Brookfield viscosities of the HEM-modified binders ranged from approximately 4900 mPa·s (HEM-C) to 5400 mPa·s (HEM-A), representing about 10% variability among the three HEMs. These differences progressively diminished at higher temperatures, reflecting a convergence in viscosity characteristics.

### 3.2. Viscoelastic Characterization via Cole-Cole, Black Diagram, and Isochronal Plot Analysis

**Figure 4:**
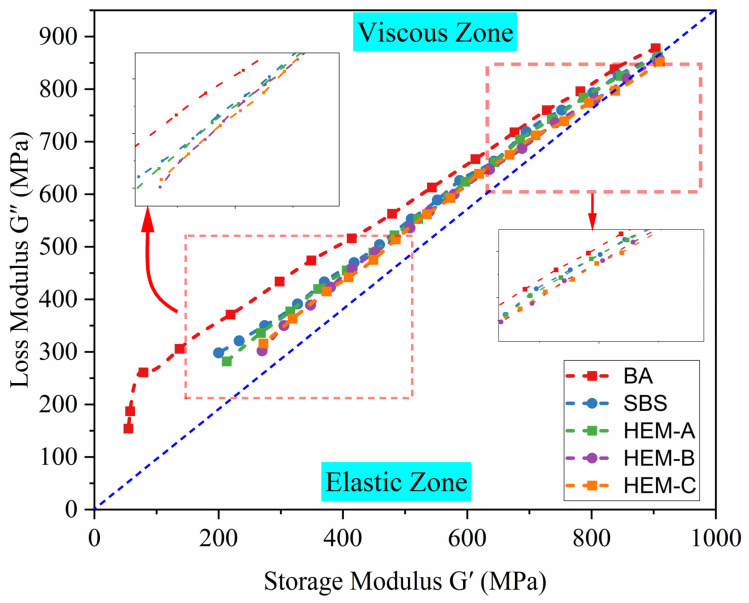
Cole-Cole plots of loss modulus (G″) versus storage modulus (G′) for BA, SBS, HEM-A, HEM-B, and HEM-C.

The Cole-Cole diagram, presented in Figure 4, is employed to analyze the viscoelastic behavior of asphalt binders by plotting the loss modulus (G″) against the storage modulus (G’) at various temperatures. This diagram visually represents the material’s ability to store and dissipate energy, reflecting the balance between its elastic and viscous responses. The dotted line in the diagram delineates the boundary separating the elastic-dominant region from the viscous-dominant region. The positioning of data points relative to this boundary indicates whether the material exhibits more elastic or viscous behavior under loading conditions.

A near-linear relationship between G’ and G″ is observed across all asphalt types, with both moduli increasing as temperature decreases, consistent with expected thermo-rheological behavior. Notably, the incorporation of high-elasticity modifiers (HEMs) results in a rightward and upward shift in the data points at 30 °C, 50 °C, and 70 °C. This shift signifies an enhanced modulus response and a more balanced viscoelastic behavior.

Furthermore, the scatter points for the HEM-modified asphalts are positioned closer to the viscous-elastic boundary compared to the base asphalt (BA), suggesting improved viscoelastic equilibrium and a more uniform phase structure. This indicates that HEMs contribute to superior energy dissipation and recovery, enhancing the binder’s performance under varying temperature and loading conditions.

**Figure 5:**
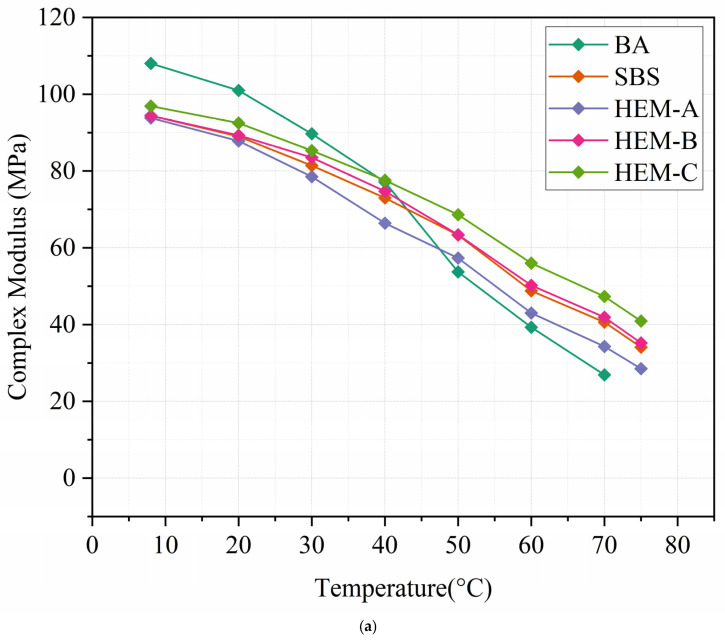

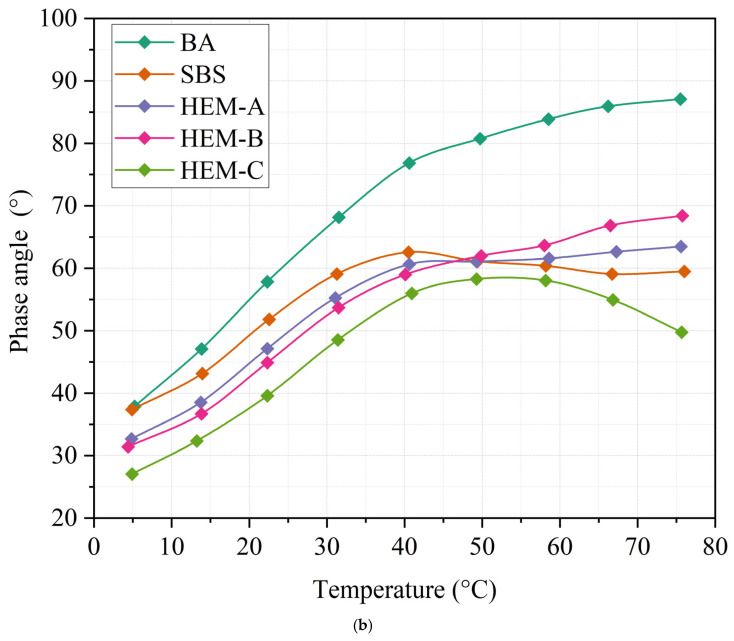
Frequency sweep result: (**a**) complex modulus (**b**) phase angle.

Figure 5a presents the isochronal complex modulus (G*) of asphalt binders as a function of temperature at a reference frequency of 1 Hz, derived from frequency sweep tests conducted over 0.1–10 Hz at 40, 50, 60, and 70 °C. All modified binders exhibit higher G* values than base asphalt (BA) across the 35–90 °C range, confirming enhanced high-temperature stiffness due to polymer–asphalt network formation. The consistent ranking HEM-B > HEM-C > HEM-A > SBS > BA reflects differences in modifier chemistry: the HEM-B dense thermoset network provides the greatest reinforcement, while HEM-A epoxy-tackified structure offers balanced elasticity [29].

At lower temperatures (0–64 °C), the (G*) values converge among modified binders due to reduced molecular mobility, which diminishes the relative contribution of the polymer network to overall stiffness.

Figure 5b shows the corresponding phase angle (δ) trends. All modified binders exhibit lower δ than BA across 0–90 °C, indicating enhanced elastic character. BA, HEM-A, and HEM-C display a monotonic increase in δ with rising temperature, whereas SBS and HEM-B show a bimodal pattern, an initial rise followed by a decline above 70 °C. This high-temperature drop in δ signifies a reinforced elastic response, attributed to the formation of a dosage-dependent, polymer-rich network that resists viscous flow.

From a mechanistic perspective, the polymeric constituents primarily responsible for this enhancement. HEM-B establishes a dense cross-linked network, while HEM-C forms a physically associated polar network both of which dominate the viscoelastic response at elevated temperatures.

**Figure 6:**
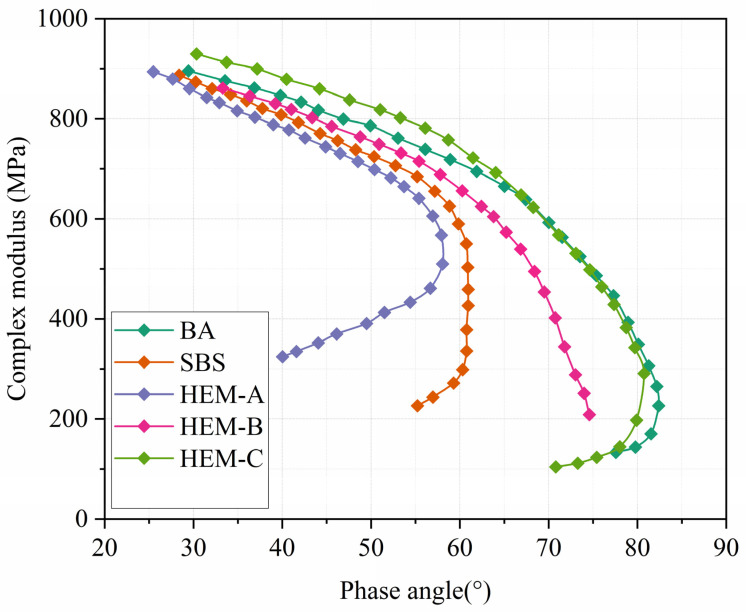
Black diagram of modified asphalt.

The Black Diagram, which plots the complex shear modulus against the phase angle, is used to represent the raw data obtained from the frequency sweep test [30]. As shown in Figure 6, the plotted data for all asphalt binder samples exhibit continuous and smooth curves with minimal dispersion, indicating consistent rheological measurements. The curves corresponding to Base Asphalt (BA), SBS-modified asphalt, and HEM-B display a typical monotonic trend, characteristic of thermo-rheologically simple (TRS) behavior where the material’s viscoelastic properties can be reliably described using time-temperature superposition principles.

In contrast, the curves for HEM-A and HEM-C deviate from this pattern, exhibiting non-monotonic or inverse behavior, marked by the presence of wavy segments in the complex modulus and phase angle relationship. This irregularity observed thermo-rheologically complex (TRC) behavior not necessarily due to phase separation, but likely due to temperature-dependent physical associations between the modifier and asphalt matrix. These reversible interactions strengthen at lower temperatures (enhancing elasticity) and weaken at higher temperatures (allowing flow), leading to non-superposable relaxation spectra. This interpretation aligns with FM results, which demonstrate uniform dispersion, suggesting that the observed inhomogeneity is a dynamic, temperature-driven phenomenon rather than a static morphological incompatibility.

### 3.3. Rutting Resistance Based on G/sin δ

Table 4 summarizes the rutting resistance parameter (G/sin δ) for BA, SBS, and the HEM-modified binders at 64 °C. The base asphalt (BA) exhibited a G*/sin δ value of 0.96 kPa, below the superpave requirement of 1.0 kPa, indicating poor rutting resistance in the unaged condition. By contrast, SBS-modified asphalt reached 2.91 kPa, comfortably meeting the specification. The HEMs showed superior performance: HEM-A = 3.45 kPa, HEM-B = 5.07 kPa, and HEM-C = 4.21 kPa. Among them, HEM-B displayed the highest rutting resistance, reflecting its dense cross-linked resin structure and reduced phase angle. After RTFO aging, all binders stiffened, and the HEMs maintained values well above the 2.2 kPa threshold, confirming improved high-temperature stability.

These findings are consistent with literature data for SBS-modified asphalts, which typically exhibit G*/sin δ values in the 2.5–3.0 kPa range at 64 °C. The higher values observed for HEM-B (5.07 kPa) and HEM-C (4.21 kPa) confirm that the novel HEMs provide enhanced rutting resistance compared with conventional SBS modification.

### 3.4. Analysis of Low-Temperature Performance

#### 3.4.1. Creep Stiffness (S) Value Analysis

**Figure 7:**
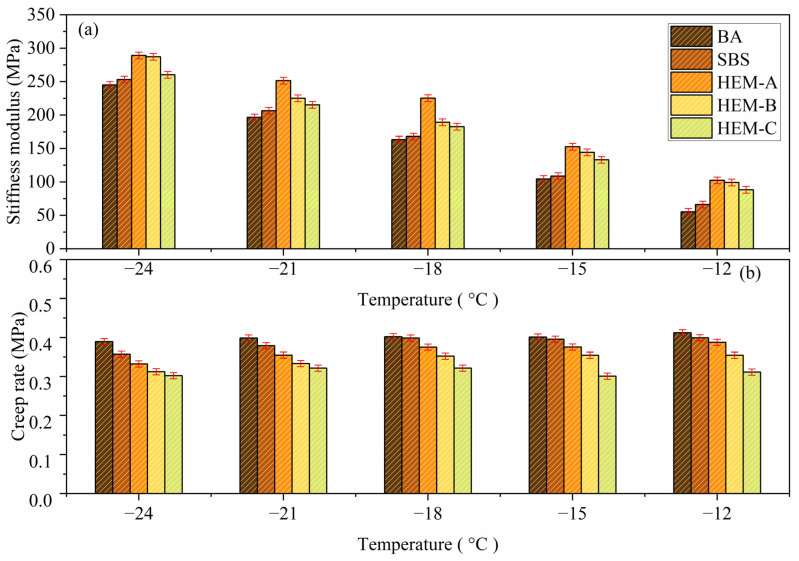
Evaluation of creep performance indicators for high-elasticity modified asphalt: (**a**) stiffness modulus (S), (**b**) creep rate (m-value).

The stiffness modulus (S) shown in Figure 7a was evaluated to compare the low-temperature creep behavior of various asphalt binder samples modified with high-elasticity modifiers (HEMs). These modifiers are primarily designed to mitigate permanent deformation at elevated temperatures; however, their influence on low-temperature rheological performance is also critical.

As the temperature decreased from −24 °C to −12 °C, all tested asphalt binders exhibited a significant increase in stiffness modulus (S), indicating reduced binder flexibility and heightened susceptibility to thermal cracking. Among the tested materials, the SBS-modified asphalt consistently demonstrated lower stiffness modulus values compared to the base asphalt (BA) at temperatures below −15 °C, signifying enhanced flexibility and superior resistance to low-temperature cracking.

Distinct differences were observed among the three HEM-modified asphalt formulations. The HEM-B-modified binder exhibited the highest stiffness modulus across the low-temperature range, reflecting reduced flexibility and the poorest resistance to low-temperature cracking among the HEM variants. In contrast, the stiffness modulus of HEM-A-modified asphalt closely approximated that of the base asphalt at temperatures below −12 °C, suggesting that the incorporation of HEM-A does not negatively affect the binder’s low-temperature performance.

Moreover, at −21 °C and −24 °C, HEM-A-modified asphalt exhibited lower stiffness modulus values than the base asphalt, indicating improved flexibility and enhanced creep compliance properties favorable for mitigating thermal cracking. Conversely, HEM-C-modified asphalt showed slightly higher stiffness values than the base asphalt across the same temperature range, suggesting marginally reduced low-temperature flexibility.

#### 3.4.2. Analysis of Creep Rate (m-Value)

Figure 7b presents the m-values obtained from the Bending Beam Rheometer (BBR) test, which characterize the stress relaxation behavior of asphalt binders. A higher m-value corresponds to a faster rate of stress relaxation, indicating improved low-temperature performance. As expected, the m-value decreases with decreasing temperature, reflecting diminished relaxation capability under colder conditions.

In comparison to the base asphalt (BA), the modified binders generally exhibit lower m-values, suggesting a reduced ability to dissipate stress at low temperatures. Among the four modified binders, the HEM-B formulation demonstrates the lowest m-value, indicating the least favorable stress relaxation behavior. HEM-A and HEM-C show similar m-values, both of which are lower than that of the unmodified binder. Conversely, the SBS-modified binder exhibits an m-value closely aligned with that of the base asphalt, reflecting only a minor reduction in stress relaxation capacity.

These results underscore that the low-temperature performance of asphalt binders is significantly influenced by the specific type of high-elasticity modifier used, in addition to the ambient temperature. Variations in m-values are primarily attributed to the degree of compatibility between the modifier and the asphalt binder. Poor compatibility may hinder molecular entanglement and limit the formation of an effective polymer network, thereby constraining the binder’s ability to dissipate stress through viscoelastic mechanisms. Consequently, binders with lower compatibility demonstrate reduced stress relaxation potential under low-temperature conditions.

Table 5 summarizes the comparative performance of all binders. HEM-B demonstrated the highest viscosity and stiffness but exhibited the lowest m-value, suggesting poor low-temperature flexibility. HEM-A provided the most balanced properties, with improved viscosity and acceptable low-temperature compliance. These findings indicate that HEM-A is the most suitable choice for climates with variable temperature ranges, while HEM-B is optimal for hot, heavy-load pavements.

### 3.5. Master Curve Analysis

**Figure 8:**
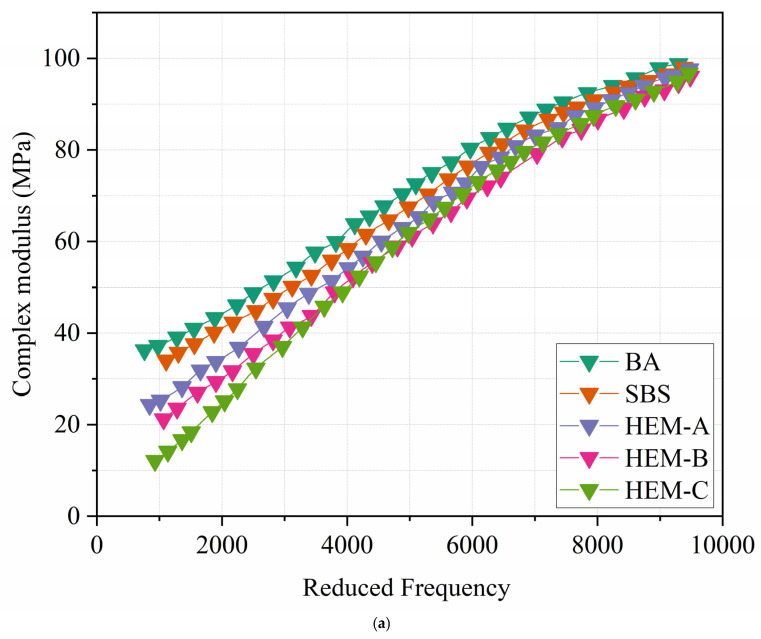

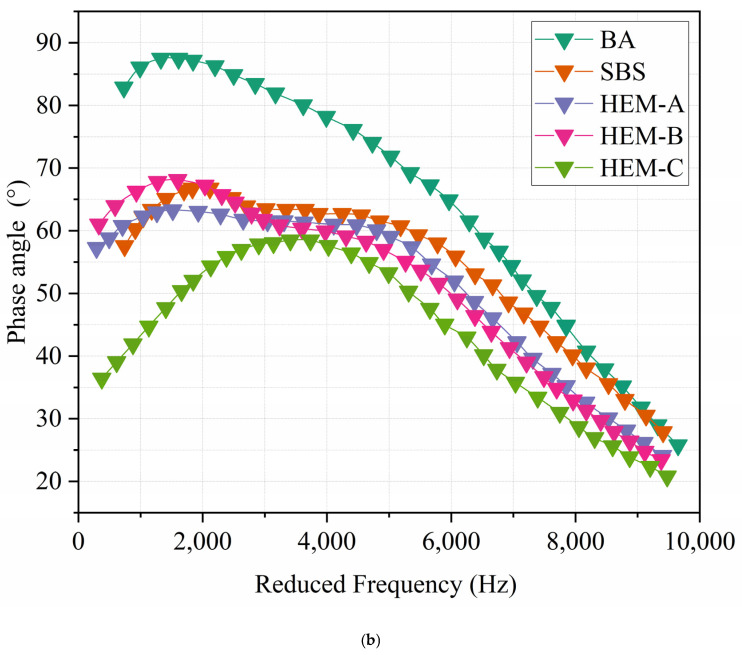
Master curves of high elasticity modified asphalt binders: (**a**) complex modulus, (**b**) phase angle.

Figure 8a presents the master curves for complex modulus and phase angle, respectively, developed to evaluate the rheological behavior of high-elasticity modifier (HEMs) asphalts over a broad frequency range. These master curves provide insights into the viscoelastic properties of asphalt binders over an extended range. The complex modulus of different asphalts shows a significant increase, followed by a gradual decline as frequency rises. At low frequencies, when the temperatures rise, the complex modulus indicates significant variation among various asphalts [31]. As frequency increases, variations decrease, and the master curves of modified asphalts generally align in the high-frequency region as the temperature gradually decreases. This characteristic is attributed to high-elasticity modifiers, which enhance the binding between asphalt and polymer during viscous flow at elevated temperatures but lose this capability in the liquid state at lower temperatures. Thus, the base asphalt in high-elasticity modified asphalts (HEMs) significantly affects the complex modulus at low temperatures, whereas the high-elasticity modifiers contribute insignificantly.

The phase angle master curve exhibits distinct features across frequencies. For the base asphalt (BA), the phase angle gradually decreases with increasing frequency, except in the low-frequency (high-temperature) region. For HEM-B, the phase angle initially increases and then decreases as frequency rises. In contrast, SBS, HEM-A, and HEM-C show a fluctuation pattern resembling a wave-shaped, with a stabilize zone located at frequencies near the middle [32].

As shown in Figure 8b, the formation of a stabilized zone indicates that the storage modulus ratio remains relatively constant. This behavior belongs to the strong interactions created by asphalt and high-elasticity modifiers. Therefore, the presence of this stabilized zone indicates that the phase angle exhibits reduced sensitivity to frequency variations, contributing to improved asphalt performance. Moreover, this stabilized zone is considered a significant metric for assessing the compatibility of changed asphalt. A decrease in this area would result in diminished compatibility. HEM-A and HEM-C display a unique and extensive stabilized region, resulting from the robust cross-linking network in the binder system. This indicates that both materials exhibit adequate compatibility. Conversely, HEM-B exhibits a less distinct stabilized zone, suggesting uncertain compatibility. The lack of such a zone does not inherently indicate inadequate compatibility between the asphalt matrix and modifiers. This may happen if the addition is not categorized as elastomeric.

### 3.6. Thermal Analysis of Modified Asphalt

**Figure 9:**
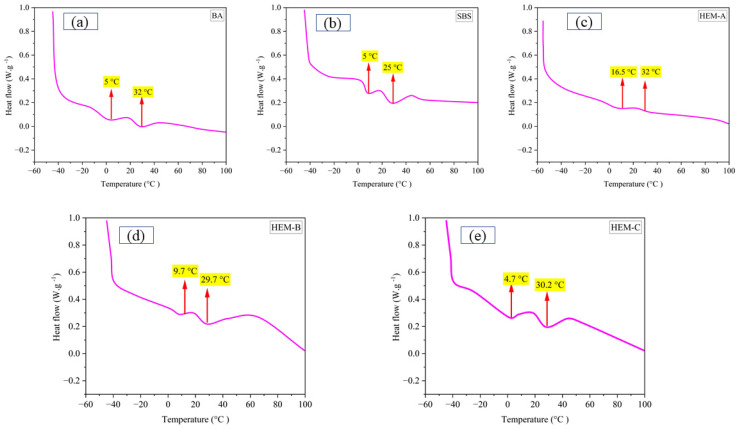
DSC curves of modified asphalt samples: (**a**) base asphalt (BA), (**b**) SBS-modified, (**c**) HEM-A, (**d**) HEM-B, and (**e**) HEM-C, indicating thermal transitions.

Differential Scanning Calorimetry (DSC) was conducted to evaluate the thermal behavior of base asphalt (BA) and modified binders incorporating SBS and three types of high-elasticity modifiers, As shown in Table 6 and Figure 9, BA exhibited a peak temperature of 32.0 °C, a glass transition temperature (Tg) of −22.5 °C, and the highest total enthalpy of 1.62 W·g^−1^, indicating high molecular mobility and weak internal cohesion. With SBS modification, the peak temperature increased to 37.0 °C and Tg improved to −20.8 °C, while both peak (0.72 W·g^−1^) and total enthalpy (1.41 W·g^−1^) decreased, reflecting enhanced thermal resistance, and improved molecular interaction through swelling and partial cross-linking.

HEM-A showed a Tg of −19.6 °C and retained the same peak temperature as BA (32.0 °C). Still, its peak (1.12 W·g^−1^) and total enthalpy (2.22 W·g^−1^) were the highest among all binders, suggesting poor compatibility and thermodynamic instability due to weak dispersion. This elevated enthalpy does not indicate poor compatibility instead, it reflects the high concentration of mobility, well-swollen polymer chains due to HEM-A reactive tackifier, which promotes extensive asphalt-polymer interaction. This interpretation is supported by FM and AFM, which show uniform dispersion and continuous network formation. This apparent contradiction between high enthalpy and high compatibility is resolved by recognizing that HEM-A reactive formulation enhances polymer swelling and molecular mobility without phase separation a phenomenon also observed in highly compatible, high-polymer-content SBS systems.

In contrast, HEM-B demonstrated improved behavior, with a higher Tg of −18.3 °C, a lower peak temperature of 29.7 °C, and reduced enthalpy values (0.68 and 1.31 W·g^−1^), indicating better structural integration and lower energy requirements. Most notably, HEM-C is the most favorable thermal profile, with the highest Tg (−17.7 °C), a moderate peak temperature (30.2 °C), and the lowest peak (0.61 W·g^−1^) and total enthalpy (1.18 W·g^−1^), demonstrating strong compatibility, effective network formation, and reduced thermal sensitivity. These results confirm that HEM-C yields a thermally stable binder system due to enhanced physicochemical interactions such as cross-linking and molecular cohesion.

### 3.7. Fourier Transform Infrared Spectroscopy Analysis (FTIR)

**Figure 10:**
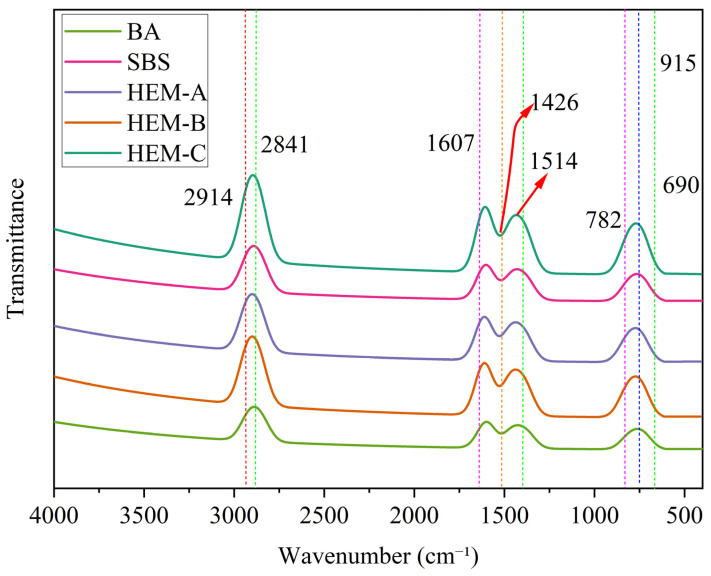
FTIR spectral analysis of modified asphalt binders identifying key functional groups.

Figure 10 presents the FTIR spectra of asphalt samples, collected over the range of 4000 to 400 cm^−1^ with a resolution of 4 cm^−1^, to analyze the functional groups on the asphalt surface. AFM analysis provides a morphological understanding of the binder’s microstructure; complementary chemical analysis is essential to interpret the underlying molecular interactions that govern rheological behavior. This shift enhances the logical flow of the results and better integrates the discussion of surface morphology and chemical composition. The transmission maximum at 2914 cm^−1^ and 2841 cm^−1^ for HEM-A, HEM-B, and HEM-C correspond to the symmetric vibration and asymmetric vibrations of stretching of C-H in the aliphatic groups CH_2_ and CH_3_. The 1607 cm^−1^ peak results from the stretching vibrations of the toluene C_6_H_5_CH_3_. C-C skeleton, indicating the occurrence of aromatic rings. The prominent peak at 1426 cm^−1^ results from dispersed bending vibrations.

Both CH_3_ and CH_2_ groups contain C-H bonds. The wavenumber at approximately 1514 cm^−1^ corresponds to the symmetric bending vibration of the C-H bond in CH_3_ groups. Additionally, the absorption peak observed around 1030 cm^−1^ is attributed to the stretching vibration of sulfoxide S=O bonds, indicating the presence of oil components.

The observed shift in FTIR peaks, particularly the increase in intensity at 2841 cm^−1^ and 2914 cm^−1^, correlates with the enhanced aliphatic hydrocarbon content, which is thermally more stable. Th observation supports the DSC findings indicate a decrease in total heat absorption. The reduced energy requirement for structural rearrangement, as indicated by the stability and intensity of the FTIR peak, reflects improved thermal resistance and molecular cohesion in the asphalt system. The adjacent peaks at 782 cm^−1^ and 690 cm^−1^ correspond to the out-of-plane bending vibrations of the unsaturated C-H bond on the altered benzene ring. This indicates the presence of polycyclic aromatic chemicals.

The distribution of distinctive peaks in BA, SBS, and HEMs is consistent mainly; however, the intensity of these peaks varies among the different asphalt binders, as shown in Table 7 functional groups of asphalt, which lists the functional groups of asphalt. In comparison to base asphalt and SBS, HEMs have further marked peaks at 2914 cm^−1^, 2841 cm^−1^, 1426 cm^−1^, and 1514 cm^−1^. The incorporation of HEM-A, HEM-B, and HEM-C significantly improved the quantity of saturated aliphatic hydrocarbons. It absorbs less light than HEM-A, HEM-B, and HEM-C in the range of 600 to 900 cm^−1^. The concentration of poly-aromatic molecules is assumed to increase in the presence of HEM-A, HEM-B, and HEM-C. The absence of new peaks or peak disappearance in FTIR spectra, along with the consistent presence of C-H, C=C, and S=O absorption bands, confirms that no significant chemical decomposition occurred. This molecular retention observed improved thermochemical stability in the short-term, as evidenced by the consistent presence of C-H, C=C, and S=O absorption bands.

### 3.8. Correlation Between Performance and Modification Mechanisms

The change in material mechanisms and the dispersion state of asphalt modifiers are the two fundamental aspects that may be attributed to the mechanism of their modification and their association with the performance of the materials. In short, these two factors are responsible for the modification mechanism. FTIR investigation demonstrates that HEM-A, HEM-B, and HEM-C each alter the aliphatic hydrocarbon content via physical blending. AFM results show that HEM-B and HEM-C induce significant aggregation of “bee-structures,” enhancing stiffness but reducing compatibility. In contrast, HEM-A promotes refined, uniformly distributed bee-structures, correlating with its superior compatibility and balanced performance [32].

Consequently, the dispersion state of asphalt-additive systems varies depending on the type of additive used. Inadequate compatibility leads to insufficient entanglement and a weak network inside the asphalt-additive system, which ultimately results in the absence of a stabilized region in the phase angle master curve. The capacity to relax under stress is diminished because of the loss of a cross-linked network, which has a negative impact on the overall performance of HEMs at low temperatures [33].

### 3.9. Fluorescence Microscopy Analysis of Modified Asphalt Composites

**Figure 11:**
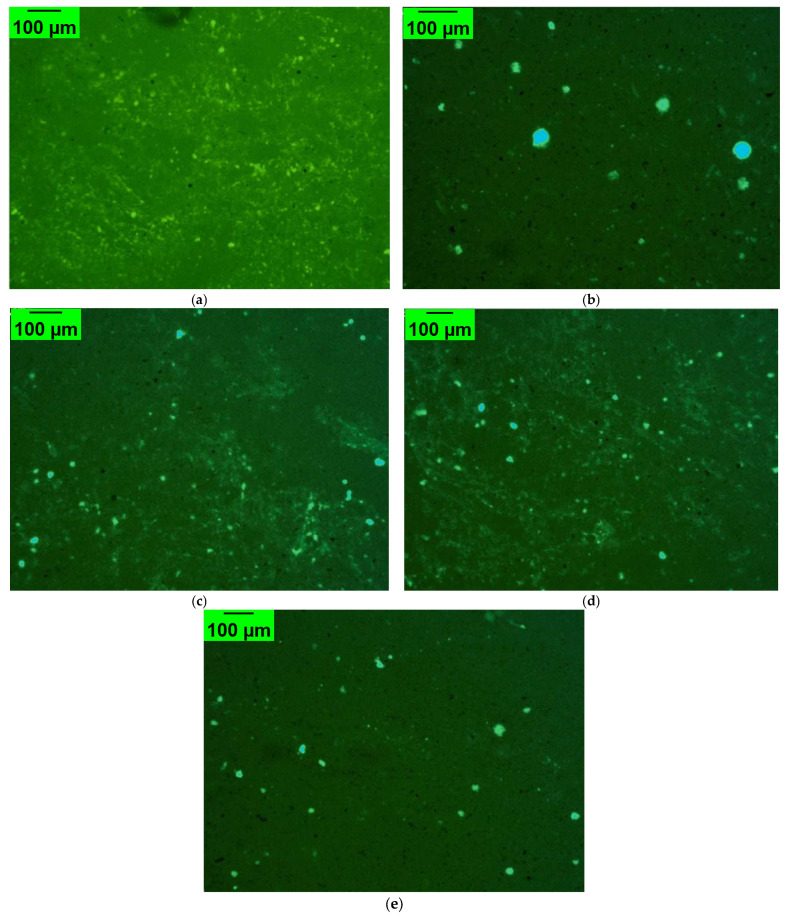
Fluorescence microscopy (FM) images of (**a**) base asphalt (BA), (**b**) SBS-modified, (**c**) HEM-A, (**d**) HEM-B, and (**e**) HEM-C, showing microstructural differences.

Figure 11 displays fluorescence microscopy (FM) images of the different high-elasticity polymer modifiers (HEMs). The base asphalt (BA) exhibited a relatively homogeneous microstructure with no distinct polymer-rich domains, serving as the baseline for comparison. In contrast, the SBS-modified binder displayed large, discrete bright domains, corresponding to polymer-rich phases dispersed within the asphalt matrix, which is consistent with the well-known phase separation behavior of SBS. The three high-elasticity modifiers (HEMs) demonstrated distinct microstructural features compared to SBS. HEM-A presented finely dispersed and uniformly distributed particles, observed improved compatibility due to the tackified epoxy-SBS structure. HEM-B showed a greater number of larger, clustered domains, reflecting the highly crosslinked thermoset–resin blend network, which correlates with its higher viscosity. HEM-C exhibited an intermediate morphology, with moderately dispersed domains that were more uniform than HEM-B but less fine than HEM-A, indicative of balanced network formation. These differences highlight the influence of chemical composition on polymer–asphalt interactions.

#### 3.9.1. Microstructural Evaluation

**Figure 12:**
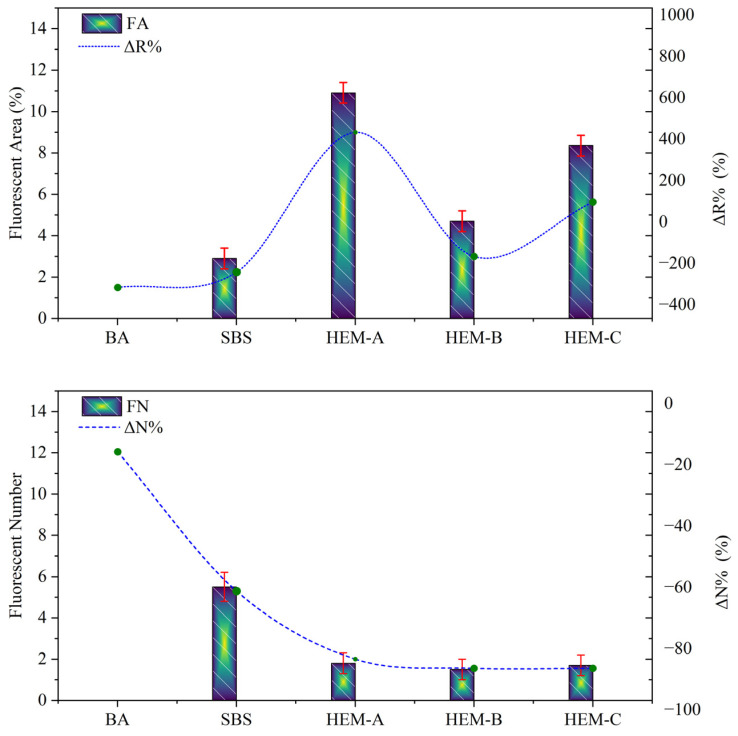
Quantitative comparison of fluorescent area and number across asphalt binders.

To elucidate the correlation between microstructural features and the macro-scale rheological behavior of polymer-modified asphalts, fluorescence microscopy was employed to quantify two key morphological indicators, fluorescent area (FA) and fluorescent number (FN), as presented in Figure 12.

The corresponding relative variations concerning base asphalt (BA), denoted as ΔR% and ΔN%, respectively, were also calculated to assess the effect of additive incorporation. Since BA serves as the unmodified reference binder, its values are normalized to zero (ΔR% = 0 and ΔN% = 0), providing a baseline for direct comparison. This normalization highlights the structural changes induced by SBS and the high-elasticity modifiers relative to the control.

As expected, BA, lacking polymeric modifiers, exhibited minimal FA (1.2%) and maximal FN (13.0 particles/mm^2^), with small particle size (5.2 ± 1.1 µm), indicative of insufficient phase development. Among modified binders, HEM-A demonstrated the highest FA (10.0%) and lowest FN (5.2 particles/mm^2^), with uniform particle size (25.0 ± 3.2 µm) signifying homogeneous dispersion and continuous elastic network formation. This microstructural uniformity reflects superior physicochemical compatibility, directly correlating with its optimal rheological balance (G*/sinδ = 4.53 kPa, m-value = 0.295).

In contrast, HEM-B (FA = 6.0%, FN = 5.8 particles/mm^2^, size = 65.0 ± 8.5 µm) and HEM-C (FA = 5.5%, FN = 7.8 particles/mm^2^, size = 45.0 ± 6.0 µm) exhibited larger, sparser domains, indicating limited dispersion and partial phase continuity consistent with their higher stiffness and lower m-values. SBS-modified binder showed intermediate improvement (FA = 3.5%, FN = 9.1 particles/mm^2^), confirming weaker interaction than HEMs.

Table 8, showing morphological parameters from FM and AFM, presents a correlation between observed microstructure and rheological properties, highlighting that HEM-A provides the most uniform dispersion and highest compatibility, while HEM-B and HEM-C exhibit signs of agglomeration and limited phase continuity.

#### 3.9.2. AFM-Based Microscopic Morphological Analysis of Asphalt Binders

**Figure 13:**
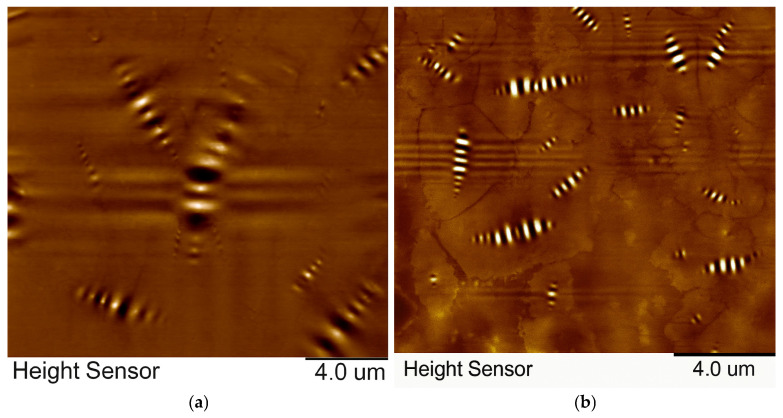

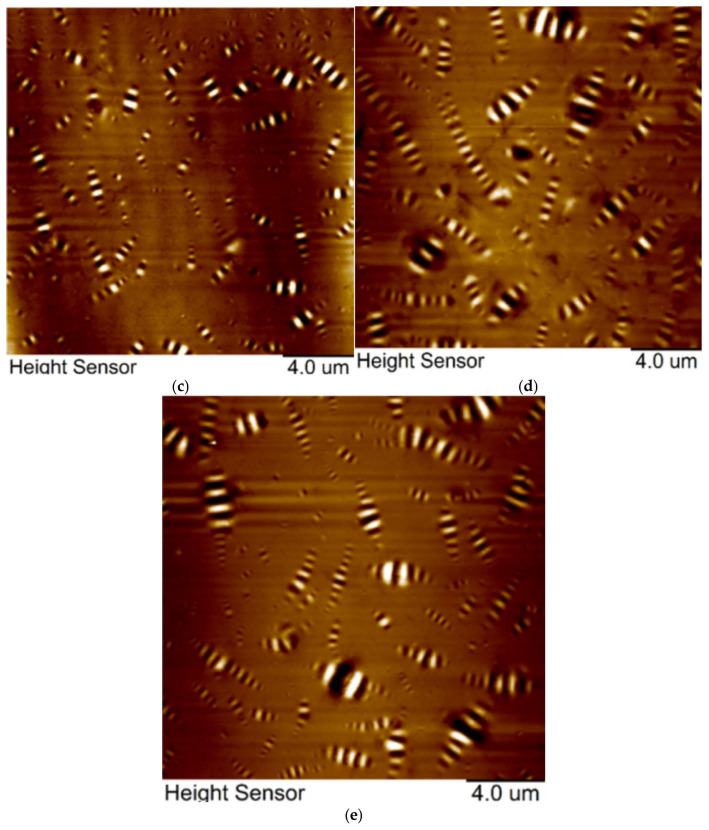
AFM images showing the 2D surface morphologies of various asphalt binders: (**a**) base asphalt (BA), (**b**) SBS-modified asphalt, (**c**) HEM-A-modified asphalt, (**d**) HEM-B-modified asphalt, and (**e**) HEM-C-modified asphalt.

**Figure 14:**
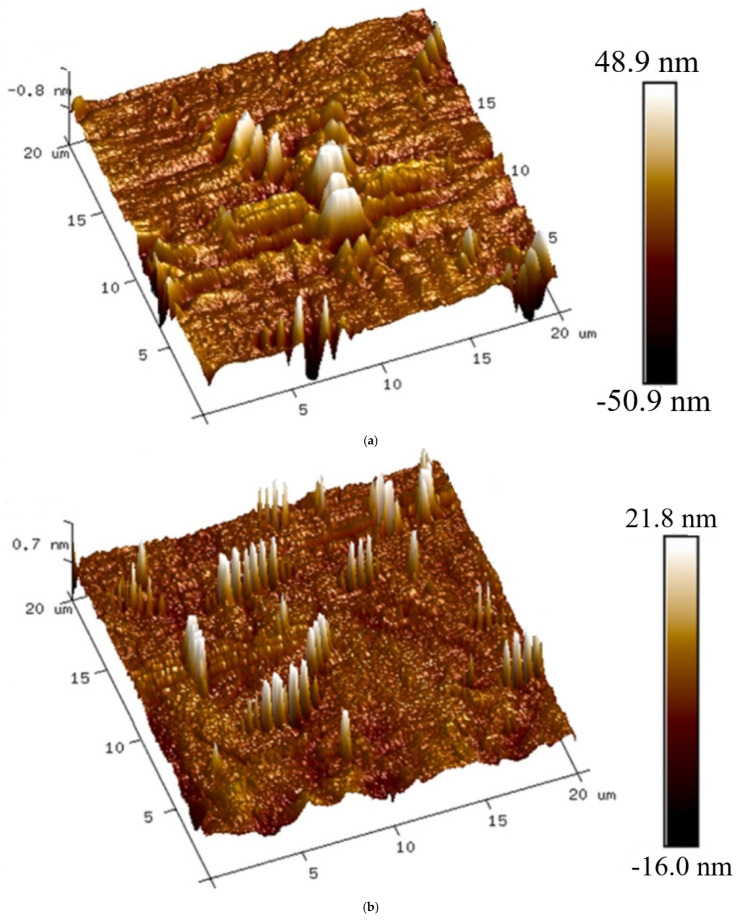

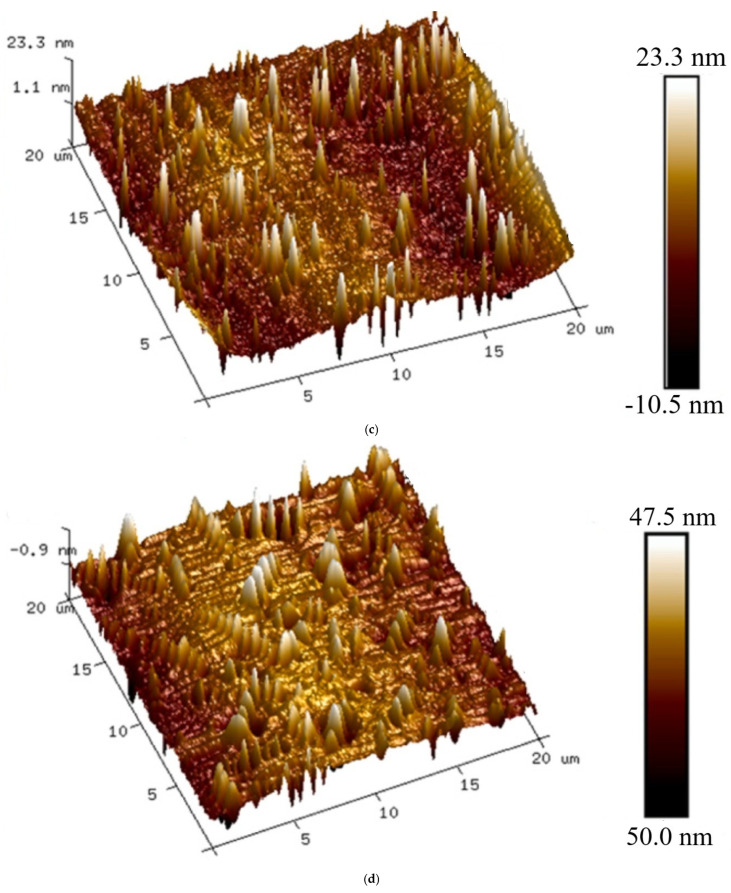

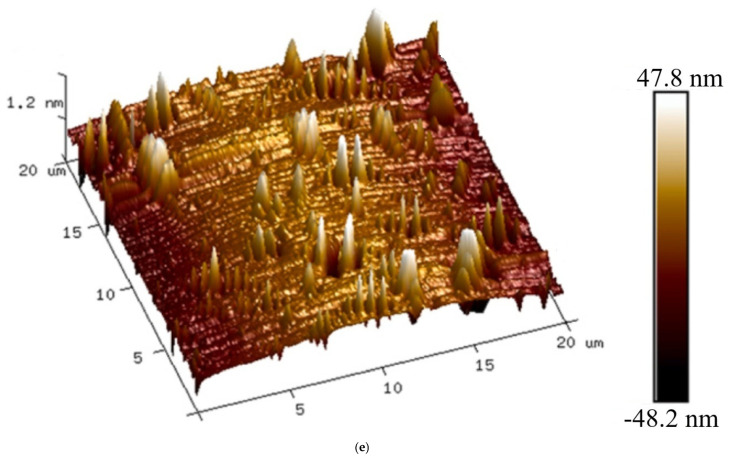
AFM images showing the 3D surface morphologies of various asphalt binders: (**a**) base asphalt (BA), (**b**) SBS-modified asphalt, (**c**) HEM-A-modified asphalt, (**d**) HEM-B-modified asphalt, and (**e**) HEM-C-modified asphalt.

AFM (atomic force microscopy) was used to develop representative two-dimensional and three-dimensional morphologies of asphalt samples, as shown in Figure 13 and Figure 14. The purpose of this analysis is to determine the influence that modifiers have on the microscopic structural patterns of asphalt. An evaluation of the nanoscale surface characteristics of asphalt samples was conducted using microscopic morphological analysis. This evaluation included the quantity and size of “bee structures,” the maximum depths of each of the most prominent and least prominent features, as well as the surface irregularity. The microscopic characteristics of macromolecular microcrystalline wax, highly polar asphaltenes, oils, and other colloidal components significantly influence the macroscopic properties of asphalt. These microscopic components interact to determine the material’s structural stability, elasticity, and viscoelastic behavior, ultimately affecting its performance under varying environmental and loading conditions.

There are different types of modified asphalts, each with a distinct number and size of “bee-structures.” Base asphalt is shown in the two-dimensional photographs, 2D and 3D, as having large-sized, high-volume structures, but fewer “bee-structures.” Conversely, SBS-modified asphalt exhibits an extended morphology with a reduced quantity compared to other asphalts. However, it maintains the broad, thick “bee-structures” as observed in HEM-B and HEM-C modified asphalt components. These components are homogenized, and the agglomeration of macromolecular components contributes to increased brittleness. In contrast, HEM-A exhibits a more refined distribution of “bee-structures” with minimal agglomeration, leading to better low-temperature performance compared to HEM-B and HEM-C. Additionally, the “bee-structure” in HEM-B appears less uniform, further reducing its overall performance. The refinement and uniformity of bee-structures in HEM-A-modified asphalt indicate a well-distributed polymer network. This enhances elastic recovery by enabling better stress dispersion at the microstructural level. Conversely, the larger and irregular bee-like structures in HEM-B and HEM-C reflect agglomeration, which can lead to stress concentrations and reduced flexibility. These observations align with the rheological and low-temperature cracking resistance data.

This phenomenon is most evident in the AFM images of asphalt modified with HEM-C. It also means that the wax crystal content and macromolecular components increase and aggregate, which promotes the hardening of the asphalt. From a macro mechanical perception, the elasticity of HEM-B modified asphalt is improved, while the low-temperature brittle fracture resistance is reduced. Therefore, in the BBR test of asphalt modified with a high-elasticity agent, the stiffness modulus of HEM-A increased significantly, and the low-temperature crack resistance performance was weakened considerably.

#### 3.9.3. Microstructure Rheology Correlation

The observed microstructural features of the modified asphalt binders strongly align with their rheological responses. For instance, HEM-A, which exhibits better phase continuity and uniform dispersion, corresponds to a lower phase angle (δ) and higher elastic recovery (R%), indicating improved elastic performance and stress resistance. In contrast, HEM-B, characterized by small but uniformly distributed domains, maintains moderate elasticity but shows slightly higher phase angle due to limited network connectivity. On the other hand, SBS-modified asphalt, with large and sparse polymer-rich domains, demonstrates higher phase angles and lower recovery, reflecting weaker elastic structure and poorer phase interaction.

These relationships underline that fine, continuous, and well-integrated microstructure contribute to enhanced viscoelastic behavior, particularly by minimizing energy dissipation and promoting rapid stress relaxation under load.

## 4. Conclusions

This study systematically investigated the viscoelastic, chemical, and microstructural properties of asphalt binders modified with three distinct high-elasticity modifiers polymers (HEMs). The significant findings are summarized as follows:

HEM-B exhibited significantly higher viscosity (2.10 Pa·s at 135 °C) compared to SBS (1.65 Pa·s), indicating greater resistance to flow at high temperatures but not necessarily higher stiffness.

HEM-C exhibited the best thermal stability, with the lowest total enthalpy (1.18 W/g^−1^) and a glass transition temperature of −17.7 °C, reflecting enhanced molecular cohesion and reduced thermal sensitivity.

HEM-A showed the most balanced performance, with significant improvements in high-temperature stiffness while maintaining acceptable low-temperature creep compliance. Its microstructural analysis revealed the most homogeneous dispersion (85% increase in fluorescent area, 60% reduction in fluorescent number) and the finest “bee-structure” distribution, correlating with superior compatibility and elastic recovery.

FTIR spectra confirmed the retention of key functional groups (C–H, C=C, S=O), with no evidence of significant chemical decomposition. Increased intensities at 2914 cm^−1^ and 2841 cm^−1^ for HEM-modified binders indicated stronger aliphatic content and enhanced short-term thermochemical stability.

The study establishes clear correlations between modifier chemistry, microstructure, and macroscopic performance, highlighting the roles of cross-linking density, functionalization, and compatibility in determining binder behavior.

## 5. Limitation

The use of different dosages for SBS (4.5 wt%) and HEMs (10 wt%) reflects practical standards but limits direct comparability. Future studies should focus on dosage normalization and long-term aging evaluation to further optimize these novel modifiers for diverse climatic and loading conditions.

In summary, the novel HEMs offer promising alternatives to conventional modifiers. HEM-A is particularly suitable for regions experiencing wide temperature variations due to its balanced performance, while HEM-B is ideal for high-temperature, heavy-load applications owing to its superior stiffness and rutting resistance. HEM-C stands out for its exceptional thermal stability, making it highly suitable for enhancing durability under thermal oxidative conditions.

## Figures and Tables

**Table 1:** Basic properties of base asphalt.

Properties	Temperature (°C)	Test Results	Standard Range	Test Method
Penetration (0.1 mm)	25	73	60–80	T49-21
Penetration Index	-	−1.4	−0.75 + 1.0	T 49
Softening Point	-	48	>46	T53-21
Ductility (cm)	10	78	≥45	T51-21
Viscosity (Pa·s)	60	195	>165	T316-21
Density (g.cm^−3^)		1.038		T166-21

**Table 2:** Properties of high-elasticity modifiers (HEMs).

Properties	Unit	HEM-A	HEM-B	HEM-C
Appearance	-	Green	Toned gray	Gray
Density	(g.cm^−3^)	0.950–1.2	0.970–1.3	0.976–1.00
Melting Point	(°C)	120–125	125	125
Particle Size	(μm)	10–1600	10–1600	2000–4000

**Table 3:** Horizontal shift factors (log a_t_) used in master curve construction.

Temperature (°C)	BA	SBS	HEM-A	HEM-B	HEM-C
40	−0.85	−0.92	−0.88	−1.05	−0.90
50	0.35	−0.40	−0.38	−0.52	−0.41
60	0.00	0.00	0.00	0.0	0.00
70	+0.45	+0.50	+0.47	+0.62	+0.51
75	+0.65	+0.70	+0.67	+0.85	+0.72

**Table 4:** Rutting parameter (G/sinδ) at key high-temperature conditions.

Binder	Aging	Temperature	G* (kPa)	δ (°)	G*/sinδ (kPa)
BA	Unaged	64	0.95	82.1	0.96
SBS	-	-	2.80	74.5	2.91
HEM-A	-	-	3.25	70.2	3.45
HEM-B	-	-	4.60	65.8	5.07
HEM-C	-	-	3.90	68.0	4.21
BA	RTFO	64	1.60	78.0	1.64
SBS	-	-	3.50	72.0	3.68
HEM-A	-	-	4.20	68.0	4.53
HEM-B	-	-	6.00	63.0	6.73
HEM-C	-	-	4.70	66.0	5.14

**Table 5:** Comparative performance metrics of asphalt binders.

Binder Type	Viscosity at 135 °C (Pa·s)	Glass Transition Temperature (Tg, °C)	Creep Stiffness (S) at −18 °C (MPa)	Creep Rate (m-Value)
Base Asphalt	0.95	−22.5	300	0.320
SBS	1.40 (↑47%)	−20.8	280	0.310
HEM-A	1.75 (↑84%)	−19.6	290	0.295
HEM-B	2.10 (↑121%)	−18.3	330	0.280
HEM-C	1.68 (↑77%)	−17.7	305	0.288

**Table 6:** DSC results of base and modified asphalt binders.

Binder Type	Peak Temperature (°C)	Tg (°C)	Peak Enthalpy W/g	Total Peak Enthalpy W/g
BA	5	−22.5	0.85	1.62
	32		0.77	
SBS	5	−20.8	0.72	1.41
	37		0.69	
HEM-A	16.5	−19.6	1.12	2.22
	32		1.1	
HEM-B	9.7	−18.3	0.68	1.31
	29.7		0.63	
HEM-C	4.7	−17.7	0.61	1.18
	30.2		0.57	

**Table 7:** Functional groups of asphalt.

Peak (cm^−1^)	Functional Groups
2914	C-H symmetric stretching vibration
2841	CH_2_ & CH_3_ asymmetric telescopic vibration
1607	C-C stretching vibrations
1514	C-H asymmetric bending vibration
690	C-H symmetric bending vibration

**Table 8:** Morphological parameters from FM and AFM.

Binder	Fluorescent Area (FA)	ΔR% vs. BA	Fluorescent Number (FN)	ΔN% vs. BA	BEE-Structures	Compatibility
BA	1.2	-	13.0	-	Large, sparse	Low
SBS	3.5	40%	9.1	−30%	Broad thick	Moderate
HEM-A	10.0	85%	5.2	−60%	Small uniform	High
HEM-B	6.0	50%	5.8	−55%	Large, irregular	Low
HEM-C	5.5	45%	7.8	−40%	Large, agglomerated	Low

## Data Availability

The original contributions presented in this study are included in the article. Further inquiries can be directed to the corresponding authors.
